# Retraction: MUC1 gene silencing inhibits proliferation, invasion and migration while promoting apoptosis of oral squamous cell carcinoma cells

**DOI:** 10.1042/BSR-20182193_RET

**Published:** 2020-08-03

**Keywords:** Apoptosis, Invasion, Migration, MUC1 gene silencing, Oral squamous cell carcinoma, Proliferation

This Retraction follows an Expression of Concern relating to this article previously published by Portland Press.

This article is being retracted from *Bioscience Reports* at the request of the Editor in Chief and the Editorial Board following receipt of a notification from two readers, alerting the Editorial Board to duplication in Figure 3D, repeated features in Figure 3E, duplications and repeated features in Figures 6A, and duplications in Figure 6B.

The authors were contacted regarding the concerns raised and have not responded. Given the concerns with the Figures, the Editorial Board stands by the decision to retract.

